# Violence against women in sex work and HIV risk implications differ qualitatively by perpetrator

**DOI:** 10.1186/1471-2458-13-876

**Published:** 2013-09-23

**Authors:** Michele R Decker, Erin Pearson, Samantha L Illangasekare, Erin Clark, Susan G Sherman

**Affiliations:** 1 Department of Population, Family & Reproductive Health, Johns Hopkins Bloomberg School of Public Health, 615 N. Wolfe St., E4142, Baltimore, MD 21205, USA; 2 Center for Public Health and Human Rights, Johns Hopkins Bloomberg School of Public Health, Baltimore, MD, USA; 3 Department of Epidemiology, Johns Hopkins Bloomberg School of Public Health, Baltimore, MD, USA

**Keywords:** Violence, HIV risk, Sex work

## Abstract

**Background:**

Physical and sexual violence heighten STI/HIV risk for women in sex work. Against this backdrop, we describe the nature of abuse against women in sex work, and its STI/HIV implications, across perpetrators.

**Methods:**

Adult women involved in sex work (n = 35) in Baltimore, MD participated in an in-depth interview and brief survey.

**Results:**

Physical and sexual violence were prevalent, with 43% reporting past-month abuse. Clients were the primary perpetrators; their violence was severe, compromised women’s condom and sexual negotiation, and included forced and coerced anal intercourse. Sex work was a factor in intimate partner violence. Police abuse was largely an exploitation of power imbalances for coerced sex.

**Conclusions:**

Findings affirm the need to address physical and sexual violence, particularly that perpetrated by clients, as a social determinant of health for women in sex work, as well as a threat to safety and wellbeing, and a contextual barrier to HIV risk reduction.

## Background

Globally, women involved in sex work, often known as female sex workers (FSWs), suffer a disproportionate burden of HIV [1]. Intervention efforts have historically sought to reduce their risk through promoting HIV knowledge and reducing individual-level risk behavior, primarily condom nonuse. Yet the risk environment, or social and physical context [2], in which FSWs live and work is central to their HIV risk [3,4], in part by compromising their ability to reduce exposures to harm or enact harm-reduction behaviors. Within this broader risk context, a rapidly growing evidence base confirms the primacy of physical and sexual violence [5],with FSWs suffering alarming levels of abuse [6-9]. Consistent with findings from general populations [10-13], violence against FSWs is increasingly associated with HIV [14] as well as STI symptoms [15] and infection [6,7,14]. Condom non-use and other sexual risk behaviors [5,6,9,16], injection drug use [14], often undertaken as coping mechanism, as well as physiologic factors and genitoanal injury concomitant with sexual violence [17,18], are largely considered responsible for these patterns. The high burden of violence against FSWs, coupled with its observed impact on HIV risk and infection, has fueled growing international interest in preventing and responding to violence against FSWs.

Understanding the key perpetrators and the nature of abuse is central to our ability to prevent, respond to, and reduce the HIV-related risks associated with violence against FSWs. Yet little clarity has emerged in this area, in part because abuse perpetrators are not always specified in research with FSWs [9]. For women in the general population, it is now widely understood that intimate partners are the predominant perpetrators of physical and sexual violence [19,20] including that which results in homicide [21]. While women in sex work similarly suffer intimate partner violence, often with significant HIV risk implications [22], they are also subject to abuse from perpetrators specific to the sex work context, such as clients [7,14,23,24] and pimps or managers [6,7,25], as well as abuse and other interference from police [6,7,26]. Increasingly, research with FSWs suggests potential differences in the prevalence and nature of abuse, and HIV risk introduced, across perpetrators. In India, client violence has been found more common than intimate partner violence [6,27]. In Moscow, abuse from clients, but not pimps, heightened STI/HIV prevalence [7]. These data demonstrate the need to understand potential differences in the nature and HIV impact of abuse against FSWs, so as to optimize prevention and intervention efforts. Against this backdrop, we sought to describe the nature of physical and sexual violence and mistreatment by perpetrator type, and clarify how such experiences relate to HIV risk, among women involved in sex work in Baltimore, US.

## Methods

In-depth interviews were conducted with women in sex work in Baltimore, MD from March -August 2012. Eligible participants were women, inclusive of transwomen, ages 18 and over who had traded sex for drugs, money or a place to stay within the past three months. Recruitment was facilitated by street outreach support programs for sex workers (n = 2), as well as needle exchange programs whose female clients are often involved in sex work (n = 2), and participants were invited to recruit up to three peers into the study. Recruitment materials, including peer referral coupons, clarified the nature of the study including the substantive topic areas of experiences in sex work, violence, health concerns and HIV risk. The final sample size (n = 35) was determined based on content saturation, i.e., no new themes emerging. All participants were included in the current analysis. Sex work venues spanned drug houses, dance clubs, and internet-based, home-based, and street-based sex work.

In private spaces, participants completed informed consent, a 45–75 minute in-depth interview that explored sex work experiences via open-ended questions, and a brief multiple choice survey. Qualitative interviews were semi-structured; extensive probing elicited a narrative response in which participants described their stories. To maximize participant reflection and self-definition of experiences, no definitions were provided for physical or sexual violence during the interview. In qualitative analysis, sexual violence was interpreted broadly to include sex that was physically forced as well as that coerced under threat of abuse or other means. Non-paying partners, often referred to as ‘boyfriends’ by participants, were classified as intimate partners. The term “client” was widely recognized and universally interpreted to mean paying partners; participants sometimes referred to these individuals as “tricks”, “johns” or “dates” in qualitative interviews; particularly where the term “date” was used, interviewers clarified the nature of the relationship (e.g., paying vs. non-paying). The quantitative survey assessed participant demographics, and aspects of sex work, including working with pimps, operationally defined as “someone who you give a portion of your earnings in exchange for arranging paying clients or providing safety.” Violence was behaviorally assessed using an adaptation from the Conflict Tactics Scale 2; [28] consistency in assessment across perpetrators (i.e., clients, police, and intimate partners) optimizes comparison across these perpetrator categories. For intimate partners, operationally defined as “boyfriend or nonpaying partner”, and clients, respectively, single items assessed past-month experiences of physical violence, i.e., having been “hit, punched, slapped or otherwise physically hurt.” For intimate partners, clients, and police, respectively, single items assessed past-month sexual violence, i.e., having been “pressured or forced to have sex when you did not want to.” Participants received $40 remuneration. Interviews were audio-recorded and transcribed verbatim.

All procedures were approved by the Institutional Review Board of Johns Hopkins Bloomberg School of Public Health. Several additional protections were implemented, consistent with ethical guidelines for violence-related research [29]. All interviewers were graduate-level, with training and experience in qualitative research as well as working with abuse survivors. Participants were carefully monitored for distress; interviewers periodically asked participants how they were feeling and provided ongoing validation. At the conclusion, all participants were screened for distress and were provided with a discreet resource sheet documenting local support services.

### Analysis

Qualitative analyses were guided by Grounded Theory [30,31] to maximize discovery and allow themes to emerge naturally from the data. We used an iterative process to identify mutually exclusive codes or themes across transcripts. To inform an initial coding scheme, two members of the research team independently read all of the interviews, and four members of the research team read and hand-memoed a set of five transcripts. After discussion and iteration, this initial structure was applied to a set of eight additional transcripts, whereby sets of coder pairs independently coded each transcript, then met to review codes, discuss emergent themes, and identify any differences in interpretation. Through this process, coders met on completion of dual coding each transcripts, approximately 1–2 times weekly, to establish inter-coder agreement via a standard, discussion-based process of identifying disagreements on codes, standard issues, including redundant codes, vague code definitions, lack of mutual exclusivity, and lack of shared understanding, were iteratively identified and resolved [32]. After refining the meaning and relevance of codes, the remaining interview data were coded independently. Through coder meetings, held weekly and with greater frequency as needed, additional codes and sub-codes were identified, and reapplied to previous interviews as needed. Generally, codes referred to broader concepts such as client abuse, whereas emergent sub-codes, such as “client violence anal coercion/abuse”, and “partner assumed sex work without explicit disclosure” provided nuance. Inter-coder agreement was maintained through the process through ongoing discussion of the application of the coding structure; in cases of disagreement, a final decision was made by the principle investigator [33]. Current analyses report specifically on themes related to the extent and nature of violence across perpetrators. Using quantitative survey data, basic descriptive frequencies were calculated for participant demographics and experiences of abuse.

## Results

Participants ranged in age from 20 to 54 years; just over half (54.3%) were over the age of 35 (Table 1). The average age at first sex trade was 22.4, ranging from 13 to 45 years. Two participants were transgender women. Roughly consistent with the underlying racial distribution of Baltimore, 57% were African American and 37% were White. Overall, 42.9% reported some form of physical or sexual violence in the month prior to the survey. Clients were the predominant perpetrators with 14.3% endorsing physical and 20% endorsing sexual abuse, followed by intimate partners with 11.4% and 8.6% reporting physical or sexual intimate partner violence, respectively. Two participants (5.7%) had been pressured or forced to have sex with police in the past month. Qualitative findings on the nature of abuse and its HIV risk implications are discussed specific to client, partner, pimp, and police relationships, respectively.

**Table 1:** Demographic characteristics and experiences of violence among women involved in sex work (n = 35)

	
	% (n)
Age	
18-25	5.7 (2)
26-30	22.9 (8)
31-35	17.1 (6)
36-40	20.0 (7)
41+	34.3 (12)
Gender/identity	
Female	94.2 (33)
Transgender	5.7 (2)
Age at first sex trade *(mean, range)*	*(22.4, 13–45)*
Race/ethnicity*	
White	37.1 (13)
African American	57.1 (20)
Other	8.6 (3)
Sources of income other than sex trade*	
None	37.1 (13)
Job	11.4 (4)
Financial help from partner	25.7 (9)
Financial help from family or friends	22.9 (8)
Other	17.1 (6)
Works with a pimp, i.e., someone who receives a portion of earnings in exchange for arranging paying clients or providing safety	14.3 (5)
Where do you identify clients?*	
Street	62.9 (22)
Cell phone	74.3 (26)
Club or salon	42.9 (15)
Internet	17.1 (6)
Hotel	28.6 (10)
My home	8.6 (3)
They come to a house that is not mine	17.1 (6)
Someone arranges it for me	2.9 (1)
Past month client violence	
Physical violence	14.3 (5)
Sexual violence	20.0 (7)
Either physical or sexual	28.6 (10)
Past month intimate partner violence	
Physical violence	11.4 (4)
Sexual violence	8.6 (3)
Either physical or sexual	17.1 (6)
Past month sexual violence perpetrated by police	5.7 (2)
Any abuse in the past month	42.9 (15)

*not mutually exclusive.

### Client violence

Client-perpetrated physical and sexual violence was severe, and at times involved weapons such as guns or knives, and resulted in serious injury. Client violence primarily occurred in the context of negotiation of sexual acts and condom use. Clients commonly threatened abuse or became violent in response to women’s refusals for particular sexual acts, often anal sex, condom nonuse, or engaging with the client altogether.

#### Clients coercion and violence to enforce demands for sex, including anal sex

I did tell a client ‘no’ once before and I got stabbed, I was in the hospital for three days…I wasn’t out there doing it. I was on my way to 7–11 and the next thing I know I turned my back and he snatched me in the alley and he stabbed me in my back, stabbed me in the back of my neck. --51 year old African American female

The severity of the abuse coupled with perpetrators’ primary focus on securing unprotected and/or higher risk sex acts, was a powerful force in undermining women’s ability to enact safe sex. Client violence rendered women vulnerable to unprotected and high-risk sex both in the immediate encounter, as well as in future scenarios given their heightened awareness of the violence that could ensue if they refused sex or insisted on condom use.

The quote below illustrates clients’ use of guns to enforce their demands for unwanted sex, in this case anal sex.

I wound up getting shot…At the time, I wasn’t into anal sex. And that’s what he wanted and I told him, ‘No.’ He could get everything else he wanted. He was like, ‘Fuck that! You made me come too fast! I want my money back.’ He was like, ‘Well, you gone let me fuck you.’ I was like, ‘I ain’t gone let you do a motherfucking thing!’ Next thing I know he pulled out a gun and he shot me. --38 year old African American female

Clients also pressured or coerced women into unwanted anal sex by changing the terms of the initial agreement; with participants agreeing to oral or vaginal sex, and clients later attempting to pressure, coerce, or force them into anal sex. Pressure and coercion were at times enforced with physical intimidation and violence.

Like times when I had guys tell me what they want when we first get together, but then when we get to their place, it’s like, we’ll go ahead and do it, but they’d be trying to take it out and put it in my behind, and it’s like they, like, they use their body weight ‑ all that type of stuff. Like, and try to intimidate me, but I won’t let them see that I’m scared. And I’m like, “I say ‘no, no.’ That’s not what you told me you wanted. --40 year old African American female

Another participant describes agreeing to vaginal sex with a client only to be violently sodomized.

I was out here tricking and got into a vehicle, and the guy took me to [park]. It was discussed that we were going to, everything. He was going to give me $40 and we were going to exchange for sex. We got to the park, we got out of the truck, everything was good. He held my hand all the way into the park. Then he said he had to go to the bathroom, so I told him I was going to walk a few steps ahead of him, let him go to the bathroom. But when it took him a minute, I stopped. When I turned around to see what was taking him so long, he hit me and he sodomized for well over an hour. --38 year old Caucasian female

In participants’ discussion of unwanted, pressured and coerced sex, use of the term “rape” was rare, and typically reserved for discussion of physically forced sexual violence.

I’ve actually been raped where they pay me the money, and then after we got finished, he actually grabbed me around my neck and was like, ‘You know what time it is? Give me the money.’ And he said, ‘Have you ever been fucked in your ass?’ And I was like, “No.” He said, ‘Well, you’re about to experience that.’ --40 year old African American female

Client perpetrators of physical and sexual violence included both regular clients who were perceived as more safe by sex workers and therefore preferred, as well as new or unknown individuals who approached them as clients.

I was sodomized from a client that I had been dealing with for six years. That’s why you have to be careful because people’s minds slip, you may think you know them but you really don’t know them…you just want to flip? I said, “no we not,” and he said “yes you are” and that’s what happened. --51 year old African American female

Client-perpetrated sexual violence, in this case defined as rape, also occurred when clients changed the terms of agreement by expecting women to have sex with additional clients. As the following woman describes, unexpected multiple client scenarios can escalate to gang rape, including anal sex and severe injury.

I say, “No. I’m not going to do both of you all.” So he said, “You’re going to do both of us.” Because the one wanted to put his thing in my mouth. And the other one had put it in from the back and all that. And I said, “No. We aren’t going to do it like that. I don’t do that. We aren’t going to do it like that.” And he just grabbed me by my hair and threw me against the car and started talking junk and doing his hands in my face like this. And his buddy says, “Man, enough of that shit. Throw her ass down. And they pull my clothes off and raped me and all that. Then he hit me in the head with something. I can’t remember what. It was a sharp object. But it busted my head right here. And just left me dead. --42 year old African American female

#### Client coercion and violence to enforce demands for condom non-use

Much in the way that client violence undermined women’s ability to influence the types of sex they engaged in, it undermined their ability to successfully negotiate condom use. One participant described client condom refusal extending to him throwing the condom out the window after she went to some trouble to secure one from a friend. Her fear of violence prompted her to escape the situation before it escalated further.

First, he wanted me to give him oral and he didn’t have no rubber. No condom, and I was like, “You know, I ain’t putting my mouth down there.” I ended up, we drove a little further, and I saw a girlfriend of mine. So, I got a condom from her. I got the condom and, you know, I gave it to him. He threw the condom out the window. Because he was like, he didn’t want to use no condom. I was like, OK? This is not going good. I got scared. He was like, “You going to do what I want you to do.” I was like, “No, I’m not.” He’s like, “Oh, yes you are.” … we was going around a ramp, he had to slow down for a speed bump, I jumped out. And I ran. --50 year old African American female

For another participant, coercive condom non-use took the form of client condom removal while he held a gun to her head. She was subsequently sodomized, illustrating the interaction and accumulation of client violence and coercive condom practices that undermine women’s safety and ability to enact HIV prevention.

He acted like he was looking to see if somebody was coming and when he came back out he had this long gun with a silencer on it… He was like, ‘Now, suck it.’ He took the condom off and said, ‘Suck it’ or whatever…And so, the whole time while I’m performing the service on him, he just keeps hitting me in my head… with like, ‘Suck it better. Suck it better. If you don’t suck it better, I’m going to blow your brains out’… I’m sucking and sucking, and he said, ‘If you use teeth, I’m going to shoot you. If you don’t do it like you don’t enjoy it, I’m going to shoot you.’ He just kept saying it, and then he kept pulling the trigger while I was doing it… He took the gun and stuck it inside my rectum and said, ‘I should just pull this trigger while this gun is inside you. --29 year old African American transgender woman

While not quantitatively assessed, interviewers probed participants about whether or not they had ever shared their experiences or sought support. Despite the pervasiveness and severity of abuse, particularly sexual violence, few women had sought support for these experiences. In part, this lack of service seeking appeared to reflect rationalization of their vulnerability to abuse, or perception of violence as a risk of doing business.

But you know, in this lifestyle nothing’s safe, in my opinion, it’s nothing safe. --27 year old Caucasian female

Another rationalizes her vulnerability to violence as “part of the game”.

Police or guys that are planning on doing something like choke you out, take the money back, rape you. All that type of shit happens. It’s part of the game, though. I mean, I don’t know, I roll with the punches. If you’re going to be in the game, you’ve got to be realistic about it. --27 year old Caucasian female

### Intimate partner violence

Similar to client violence, both physical and sexual violence from partners was severe. It included extensive control, sexual violence, and firearms. The following story also illustrates the limited protection and support experienced by FSWs who experience partner abuse.

It was a whole bunch of domestic violence and to the end he got to where he was raping me, holding me hostage in my house, and the last time he came over, he pistol whipped me. Beat me up really bad and I was actually fighting for my life. I got the gun from him and fired a couple of shots. And I was the one that was jailed in the end. --54 year old African American female

#### Physical partner violence in response to sex work: extension of jealousy and sexual control

For many, sex work was a context for partner abuse, with women describing their partner’s discovery or affirmation of sex work as a prompt for physical abuse.

I got an email from one of the customers at work, or, a text from one of the customers at work, saying about he wanted to take me out on a date with this other girl, because he’s had this whole fantasy about having a threesome. And my boyfriend hit the roof. Like, he went ballistic. He beat the crap out of me. --34 year old Caucasian female

Abuse on confirmation of sex work occurred even when sex work may have been tacitly accepted.

[my boyfriend] found out a year ago that I was tricking. He knew but he just didn’t want to see it. And he’s a drug addict too. And he was in prison. And when he came home he said, “I hear you were on the streets.” I say, “Yeah, because I had to survive. You weren’t here to do anything for me.“ So he said, ”OK, well I understand. Well I’m home now. Why do you have to do that?” He was a little mad. He slapped me. He beat me up before. -- 42 year old African American female

#### Partner provocation to prompt sex work

Others described partner provocation so as to prompt them to engage in sex work as a means of securing income, often for drugs. Sexual violence in partner relationships can enable HIV risk much in the way it does for general populations; in addition, partner abuse appears to enable HIV risk in part through prompting sex work itself.

Yes, we fought. Because in the beginning they didn’t want me out there, but then when they seen how I was making the money, they would start arguments with me, knowing that when I argued I’m going to go outside. That would be their way of getting me to go outside so I can get some money. [Int: To support them?] Their habit and mine. -40 year old African American female

### Pimp violence

Few (n = 5) participants were actively working with pimps, operationally defined on the survey as someone who receives a portion of earnings in exchange for arranging paying clients or providing safety. Of the five, two described these individuals as boyfriends, two described them as managers, and one described him as “other”. Whether current or past, pimps were difficult to distinguish from intimate partners, as they were commonly described as boyfriends or friends, and included sexual relationships. For some, pimp relationships began as casual or serious intimate relationships that developed pimp-like dimensions as men turned their partners out for sex work to support both of them.

#### Pimp physical violence in the context of jealousy and sexual control

The following participant illustrates the potential for both unprotected sex with pimps, as well as violence in the context of jealousy and sexual control, even when pimps themselves are arranging and profiting from sex trade.

Then I ended up pregnant by him, and of course he beat on me severely. He would set me up with guys for money, and then after I would do it he would beat me up because he was jealous, because this guy would ask for me again or something and he would think something happened, or I was holding back on him. It was always something. I was eight months pregnant by him, and he kicked me down a flight of steps. We got arguing and he kicked me down a flight of steps, and my son was born stillborn. I got a birth certificate and a death certificate all in the same day. I think that was the hardest thing, one of the hardest things I’ve ever went through. --38 year old Caucasian female

#### Pimp physical violence to maintain control and ensure compliance

Those who had worked with pimps also described severe violence and forced sex work, including within the context of financial retribution. Again, the primary means of sexual risk for women involved with pimps appeared to be through clients, including being forced into sex with multiple men sequentially.

When I first moved in, I would go out [engage in sex work] when I felt like it. I would make a couple dollars. I would bring him back a vial, maybe two vials of crack. That would be about it and I stayed in the house. I paid rent from my SSD check. He never paid shit. He totally got mad one day that I paid the rent and then I caught that he wanted something and I wasn’t giving him shit. He hit me. He punched me in my face. Then I gave him everything I had and it all went downhill after that. He started bring guys to the house, making me have sex with them. One time he locked me in the closet for three days. I was pissing and shitting on myself. He would bring guys to the house. One time he brought eight different guys to the house. I had to fuck them all one after another after another after another. I went to the hospital for that. --29 year old African American female

Others were more explicitly recruited by pimps outside the context of an existing relationship. In speaking to the potential for severe violence, one such participant describes her pimp’s control, enforced with the threat of a gun, and its culmination in her purposeful arrest as a means of escaping him. Pimps exploited women’s social vulnerabilities, in the following case homelessness, as a means of establishing their control.

He wasn’t going to let me out of his sight. He had a room upstairs down the street somewhere, up over this store or something. He was always going to watch me. It was like, basically, he was kidnapping me. That’s what he was trying to say. Like, “You haven’t got anywhere to go.” She is homeless. “I got you. You aren’t going to leave me. You’re going to make that money right here in the basement, and you’re going to be with me.” … Yeah, he wanted half of it. Then he goes, “Well, you’re mine. You work for me.” I’m like, “How am I going to get away from this guy?” He had a gun and everything. He was a pimp… What I did was I went on the street, and when I saw the cops, I pulled my dress up like this, and I said, “You want a blow job,” in front of the cops so that they could arrest me. That was the only way that was going to save me from the pimp. --42 year old African American female

### Police abuse and interference

#### Police sexual coercion through exploitation of power dynamics

Overwhelmingly, the primary form of police abuse was coercive sex whereby police exploited power dynamics inherent to their relationship with sex workers. The threat of arrest, largely implicit though occasionally explicit, was sufficiently powerful to coerce sex, most often oral sex, such that overt force and physical violence from police was rarely discussed. Participants often described these scenarios as exchanging sex for their freedom.

A little bit of everything has happened to me on the street, I’ve been propositioned by police, I’ve traded my freedom and drugs from police for sex. -- 51 year old African American female.

In some cases, police were described as further flaunting their authority and exploiting these dynamics by arresting women after coercing them into the sex that would allegedly secure their freedom. These scenarios were generally described as less sexually risky in that police commonly used condoms. Yet condom use was at the discretion of police with little FSW influence, again reflecting an inherent power imbalance that undermines women’s ability to enact HIV prevention.

I’ve had sex with cops who had arrested me. After I’ve given them a blow job. They put a condom on, so you aren’t touching anything… You’ve got some of them that they use their authority to get what they want. --42 year old African American female

As this participant goes on to illustrate, the stark power imbalance between police and women in sex work reflects the illegal nature not only of sex work, but importantly the other drug-related activities that many participants were involved in.

If they catch you with a stem [crack pipe] on you or some drugs on you or something or they catch you doing something wrong or illegal sometimes they’ll let you go if you do something for them…Mostly it’s just a blowjob because it’s really quick… There’s no money transacted but it’s like damn….You’re not getting no money, no tip, no nothing, but you’re just staying free.

## Discussion

Our study confirms a high level of physical and sexual violence among women involved in sex work from a range of perpetrators, with over 40% reporting such abuse within the past month alone. Violence, particularly that from clients, undermined FSWs’ agency in HIV risk reduction. In other words, it restricted their ability to control the conditions of, including protection for, any given sexual act, thereby undermining their ability to protect themselves from STI/HIV. Findings affirm the relevance of violence as a central component of FSWs’ risk environment, i.e., that which is beyond their immediate control. By focusing on the micro risk environment [2], specifically the role of individuals, including clients, police, pimps and intimate partners, in creating harm, findings illustrate the mechanisms by which this micro environment compromises health and wellbeing. Conceptualizing abusive individuals as part of the risk environment must not detract from, but rather affirms, the clear need for accountability and consequences for these individuals, as well as interventions to modify their violent behavior. The differences in prevalence and nature of abuse by perpetrator underscore the primacy of client violence in underpinning HIV risk. The severity of abuse described herein emphasizes the need to promote safety and access to violence support services for FSWs. Our findings contextualize prior evidence linking violence with STI/HIV among FSWs [7,14] and demonstrate an urgent need to prevent and address gender-based violence to improve FSWs’ overall health and wellbeing, and mitigate HIV risk.

Consistent with past results from India [6,27], clients emerged as the primary perpetrators of violence. Moreover, client violence uniquely enabled HIV risk, with participants describing coerced condom non-use as well as overt force and coercion for anal intercourse. Our qualitative data imply that coercive dynamics likely underpin prior quantitative findings linking violence with condom non-use [6,34] and anal sex [6,7,35]. The consistency of our findings with evidence of coercion for condom negotiation [36], anal intercourse [5], and multiple unwanted clients [7,16] among sex workers in other settings underscores the global relevance of violence and coercive dynamics in heightening HIV risk for women in sex work.

Extending findings from other settings [22], intimate partner violence against FSWs was severe and often inherently linked with women’s involvement in sex work. Sex work served as a context and justification for abuse, even when abusive partners instigated or forced sex work, echoing past work that affirms sexual jealousy and control as a context for partner abuse [37]. Partner violence likely compromises women’s agency in STI/HIV risk reduction, and contributes risk, particularly if it is sustained over long periods of time. These data shed much-needed light on prior research linking intimate partner violence with both sex work and transactional sex [38,39], and caution against considering these experiences in silos. As has been previously reported [25,40,41], partners can prompt women’s uptake of, or continuation in, sex work over a continuum spanning encouragement to force; presenting challenges in distinguishing intimate partners from pimps for researchers and practitioners alike. Practitioners, including those that provide violence support services as well as HIV prevention, should be aware that individuals presented as intimate partners can also serve as pimps. Pimp relationships were also instigated through overt recruitment, which often exploited women’s underlying social vulnerabilities. Pimps generally conferred threats to safety rather than protection. Consistent with findings from other settings [7], the most immediate means of sexual risk for women with abusive partners and pimps appeared to be through clients, who were at times numerous, sequential and forced, raising concern for abrasions that may exacerbate STI/HIV transmission and underscoring the need for trauma-related support.

Few participants (n = 2) reported forced or coerced sexual activity from police on the quantitative survey, and qualitative results affirm that police abuse largely consisted of exploitation of underlying power dynamics for coercive sex with limited use of physical violence or force. These data echo findings from other settings of coerced sex with police to avoid trouble [26]. That these encounters were often described as condom-protected minimizes to some extent STI/HIV transmission concerns, however condom decisions were unilaterally made by police. Notably, police abuse was enabled not only by the illegal nature of sex work but importantly by the illegality of the substance use with which so many were engaged. That possession of illegal substances and paraphernalia exacerbates police exploitation in the form of coerced sex provides much-needed guidance on police practices, and challenges a simplistic notion that the illegality of sex work alone enables abuse. A broader lens, one that recognizes the ubiquity of substance use within this population [14,42-45], as well as the substance use-related laws that can be used against FSWs, is needed to sufficiently enable police protection and eradicate abuse.

Future research with sex workers, including that related to HIV, will benefit from clarification of abuse perpetrators. Participant descriptions of coerced and forced anal sex demonstrate the need for specificity of vaginal vs. anal sex in research, particularly in light of the transmission efficiency of anal intercourse [46]. That participants rarely described experiences of sexual violence as rape highlights the need for specific, behavioral language when assessing sexual violence, as is standard in gender-based violence surveillance in the general population [19,20]. Further work is needed to optimize assessment of police violence and sexual coercion; participants were mixed in their descriptions of choice in trading sex for freedom from police interference, which could mask the coercive or exploitative nature of the underlying power dynamics and render prevalence estimates for police abuse conservative.

Findings should be considered in light of several limitations. The small sample size, while appropriate for qualitative research, does not support population-level inferences with respect to prevalence and patterns observed. So too, it does not enable comparisons across factors such as age, race, gender identity, or location of sex work. The use of a non-probability sample may have introduced other sources of bias, for example, our use of needle exchange programs as a recruitment site may have resulted in an over-representation of substance-involved women. While generalizability is enhanced by inclusion of a variety of sex work venues, the extent to which findings generalize to other geographic settings is unknown. Despite the high level of interviewer training, social desirability and other biases may have resulted in non-candid responses.

## Conclusion

Together, findings clarify important distinctions in the nature of abuse across perpetrators, thus extending past research demonstrating significant burden of violence against women in sex work. Given the level and severity of violence, it is critical to ensure access to violence-related support services as well as safe reporting mechanisms for FSWs. There exists a clear need for trauma-informed care for FSWs that acknowledges the risks of abuse from clients, partners, police and pimps alike, as well as an understanding by violence service networks of this risk and its implications. Efforts to reduce STI/HIV for women involved in sex work should incorporate violence prevention and support; such efforts should explicitly target client violence given its prevalence and demonstrated negative impact on sexual risk.

## Competing interests

The authors declare that they have no competing interests.

## Authors’ contributions

MRD obtained funding, led study design and implementation, and drafted the manuscript. EP, SLI and EC conducted qualitative data collection and analysis, and contributed to the writing. SGS participated in the design and interpretation of findings, and helped to draft the manuscript. All authors read and approved the final manuscript.

## Pre-publication history

The pre-publication history for this paper can be accessed here:

http://www.biomedcentral.com/1471-2458/13/876/prepub
